# Pathology-Based Animal Cancer Registry of Abruzzo and Molise Regions (Central Italy): A Ten-Year Retrospective Study (2014–2023)

**DOI:** 10.3390/vetsci11110521

**Published:** 2024-10-26

**Authors:** Giovanni Di Teodoro, Francesca Cito, Romolo Salini, Marina Baffoni, Sabrina V. P. Defourny, Antonio Cocco, Nicola D’Alterio, Chiara Palmieri, Antonio Petrini

**Affiliations:** 1 Istituto Zooprofilattico Sperimentale dell’Abruzzo e del Molise “G. Caporale”, 64100 Teramo, Italy; f.cito@izs.it (F.C.); r.salini@izs.it (R.S.); m.baffoni@izs.it (M.B.); s.defourny@izs.it (S.V.P.D.); a.cocco@izs.it (A.C.); n.dalterio@izs.it (N.D.); a.petrini@izs.it (A.P.); 2 School of Veterinary Science, The University of Queensland, Gatton, QLD 4343, Australia; c.palmieri@uq.edu.au

**Keywords:** Animal Cancer Registry, comparative oncology, tumor frequency, tumor topography, tumor morphology, proportional morbidity ratios, dogs, cats, One Health

## Abstract

Pets have a crucial role in cancer research. Specifically, dogs and cats share the same environment as their owners and thus may serve as sentinels of naturally occurring tumors that are linked to the exposure to environmental hazards. Quantitative comparison of tumor types may reveal unusual cancer frequencies, providing directions for research and generation of hypotheses of cancer causation in a specific area and identification of risk factors. The aim of this study was to describe the data collected by the pathology-based animal cancer registry, managed by Istituto Zooprofilattico Sperimentale dell’Abruzzo e del Molise (IZSAM), during 10 years of activity (2014–2023) and to assess its potential epidemiological relevance. Frequencies of tumor topography and morphology in dogs and cats were described, analyzed and compared. Proportional morbidity ratios (PMRs) were calculated, taking into consideration some potential risk factors such as species, breed, sex, diet and living environment. The database comprises 5311 tumors (n = 4719 in dogs and n = 592 in cats), with a higher prevalence in females (67.3% in dogs and 61.2% in cats). The mean age at the first diagnosis of tumors was similar between sexes and slightly lower in dogs compared to cats. PMRs highlighted certain risk and “protective” factors for the development of tumors in specific topography. The risk of developing tumors of the blood and hematopoietic system (PMR = 0.44; 95% CI: 0.21–0.94), skin and subcutaneous tissues (PMR = 0.70; 95% CI: 0.61–0.80), oral cavity and pharynx (PMR = 0.60; 95% CI: 0.24–0.89), urinary organs (PMR = 0.33; 95% CI: 0.11–0.99) and bones, joints and cartilage (PMR = 0.72; 95% CI: 0.22–0.98) was lower in non-neutered male dogs than in neutered male dogs. Non-spayed female dogs had a greater risk of developing tumors of the mammary gland (PMR = 1.75; 95% CI: 1.57–1.96), female sexual organs (PMR = 2.12; 95% CI: 1.01–4.36) and respiratory system (PMR = 2.25; 95% CI: 1.55–6.74) but less risk for cutaneous and subcutaneous tissue tumors (PMR = 0.44; 95% CI: 0.38–0.51) and blood/hematopoietic system tumors (PMR = 0.47; 95% CI: 0.26–0.85) compared to spayed female dogs. Compared with mixed breed, purebred dogs had a significantly greater risk of developing mammary gland tumors (PMR = 1.36; 95% CI: 1.20–1.54) and lower risk for respiratory (PMR = 0.15; 95% CI: 0.07–0.32), gastrointestinal (PMR = 0.63; 95% CI: 0.34–0.94) and oral (PMR = 0.59; 95% CI: 0.36–0.96) neoplasia. Non-neutered male cats had a lower risk of developing skin and subcutaneous tumors (PMR = 0.68; 95% CI: 0.50–0.92) compared with neutered cats. The risk of developing skin and subcutaneous tissues tumors was higher for dogs and cats that lived mostly outdoor (PMR dogs = 1.21; 95% CI: 1.10–1.33; PMR cats = 1.18; 95% CI: 1.08–1.47), while dogs that live mainly indoor had a greater risk to develop mammary gland tumors (PMR = 0.78; 95% CI: 0.68–0.89). Results described herein highlight the fundamental role of animal cancer registration initiatives. These efforts would contribute to the possibility of conducting multicentric collaborative studies to deepen the knowledge of the epidemiology of tumors in dogs and cats from a comparative perspective, thus fulfilling the One Health approach.

## Simple Summary

Pets share the same environment as humans, making them useful for studying how environmental factors, such as pollution, toxicants and microorganisms, could contribute to cancer onset and development. To better understand tumor frequency, diagnoses, topography and related risk factors, we performed a retrospective study of tumor cases in dogs and cats in Abruzzo and Molise regions (central Italy) over a ten-year period. We analyzed over 5300 tumor cases, with the majority of cases found in dogs. The mean age at the first diagnosis of tumors was similar between sexes and slightly lower in dogs compared to cats. Female animals were the majority, and the most affected sites were the skin, mammary glands and subcutaneous tissues. Non-neutered animals and those living outdoors showed higher risks for certain types of tumors, such as malignant mammary tumors and skin and subcutaneous tumors, respectively. This study also revealed that purebred dogs had a higher risk of developing mammary tumors compared to mixed breeds. Understanding cancer epidemiology in pets could ultimately help protect both animal and human health by implementing prevention strategies that align with the One Health approach.

## 1. Introduction

Cancer is one of the most important causes of death in companion animals and humans [1,2]. Several environmental toxic substances have been proven to be important risk factors for chronic diseases and cancer, thus representing significant health risk for humans and animals. In some cases, strong scientific evidence confirmed positive associations between a specific environmental compound exposure and disease (i.e., asbestos exposure and mesothelioma, herbicides and insecticides exposure and bladder and testicular tumors) [3,4,5,6,7]. However, the cause-and-effect relationship between environmental hazards and cancer development is often very hard to demonstrate. In this regard, numerous authors suggest that human epidemiological studies should be accompanied by similar studies conducted on sentinel animals in order to prevent and overcome confounding factors and biases, such as chronic low-dose exposure, multiple exposure routes, very prolonged latency periods and non-specific health outcomes. [8].

Pets play a crucial role in comparative oncology research. Dogs and cats share the same environmental conditions as their owners, and these companion animals may serve as “sentinels” of naturally occurring tumors that are linked to the exposure to environmental carcinogens [8,9,10,11,12]. Due to their shorter lifecycle compared to humans, pets can serve as an early biological warning system for the development of spontaneous tumors and specifically environmentally driven tumors, such as those caused by toxic substances, pollution and microorganisms [9,10,11,12]. Furthermore, many spontaneous tumors in dogs and cats exhibit clinical manifestations, prognosis, pathogenesis, histological features and genetic modifications equivalent to human tumors, making them an excellent model for comparative oncology and epidemiological studies [13,14,15]. This provides an enriched population for the investigation of novel therapeutic targets, treatment modalities and environmental risk factors in oncological research [14].

These concepts are the foundations of comparative oncology, an emerging and rapidly expanding field of research aiming at studying cancer biology, cancer risk and tumor development across different species. The final purpose is to provide suitable models for advancing the understanding, diagnosis, and management of cancer in humans [13].

Cancer data are the cornerstone for prevention and control. In particular, cancer registries represent a fundamental tool that systematically collects and stores validated and comprehensive data, including patients’ demographics (sex, age, date and/or place of birth, residence) and cancer-related data such as individual tumor characteristics (location, morphology, grading, stage and behavior) [16,17]. This information is extremely useful for both clinicians and epidemiologists, providing insights that can be successfully used for health care planning and monitoring, evaluating the effectiveness of cancer screenings, highlighting potential risk factors in a specific population or geographical area and implementing preventive and control measures in humans and animals [14,17]. Moreover, effective animal cancer monitoring is essential for generating critical scientific data on the potential translational role of companion animals for comparative human studies [18,19]. This crucial epidemiological tool allows the formulation of hypotheses, facilitating the development of more accurate analytical studies to identify causal links between exposures and cancer risks [14].

Based on the type of cancer data available, three different types of cancer registries can be generated: (1) hospital-based cancer registries and (2) pathology-based cancer registries that collect all medical records from patients in a given hospital or diagnostic laboratory, respectively [20,21], and (3) population-based cancer registries that record all new cancer cases generated by hospital and pathology-based cancer registries within a well-defined population in a specific geographical location and thus are considered the gold standard in human cancer epidemiology [20,21].

In the past, animal cancer registries (ACRs) have unfortunately been sporadic and short-lived and lacked in communication and collaboration [14,22,23]. In recent years, there has been a growing interest in establishing and enhancing regional, national and international databases for animal cancer registration and to develop collaborative networks with human cancer registries [14,17].

By comparing and measuring the occurrence of tumor topographies and morphologies in cats and dogs, ACRs can provide important insights into similarities and differences in tumor occurrence, contributing to generating evidence and hypotheses to support and guide clinical and comparative research questions [14].

The aim of this study was to describe and analyze the data collected by the pathology-based bi-regional animal cancer registry (ACR), managed by Istituto Zooprofilattico Sperimentale dell’Abruzzo e del Molise (IZSAM), during ten years of activity (2014–2023) and to assess its potential epidemiological significance. In addition to providing data on frequencies, diagnoses and topographies of tumors in dogs and cats, proportional morbidity ratios were calculated in order to identify potential risk factors (breed, sex, reproductive status, prevalent diet type—homemade/commercial—and prevalent living environment—indoor/outdoor.

## 2. Materials and Methods

### 2.1. Data Source

The pathology-based Animal Cancer Registry (ACR) of Abruzzo and Molise regions (Central Italy) was established in 2014. Registry data are derived from diagnostic cases of the histopathology laboratory of Istituto Zooprofilattico Sperimentale dell’Abruzzo e del Molise (IZSAM). The activities of the ACR are supported by public funds that allow to provide free histopathological evaluation to veterinary practitioners and veterinary laboratories working in the registry’s catchment area. Veterinary practitioners were informed about the registry and invited to submit any suspected tumor (biopsies and whole-tissue samples) from dogs and cats living in these two regions. With this purpose, a specific animal cancer registry form was created to standardize the collection of suspected tumors. The form was made available to veterinary practitioners on the institutional website of IZSAM and contained information about the clients (e.g., veterinary practitioners and/or veterinary clinic/hospital), the animal’s owner (e.g., place of residence/postcode), the animal and the tumor [24]. Animal information included species, breed, microchip number, date of birth or age, sex and reproductive status, type of diet and living environment (indoor/outdoor). Tumor information included anatomical site (topography), size and gross features of the neoplasia, sampling modality (e.g., biopsy, complete excision and necropsy), date of excision, clinical stage and any related anamnestic, clinical and diagnostic information. Samples were immediately formalin fixed by the veterinary practitioners, sent to the laboratory, sectioned, routinely processed for histology and stained with Hematoxylin and Eosin (HE) for histological examination. Margin evaluation was performed at request. Immunohistochemistry (Cytokeratins, Vimentin, GFAP, Desmin, alpha-SMA, von Willebrand factor, Melan-A, S100, MUM1, CD20, CD1a, CD3, CD79a) and special stains (Toluidine Blu, PAS) were also performed with poorly differentiated tumors. In addition, since 2021, the ACR was integrated with cancer cases diagnosed during diagnostic and/or forensic routine autopsies performed at the diagnostic pathology laboratory of IZSAM on non-cancer submissions. As per Directive 2010/63/EU of the European Parliament and of the Council of 22 September 2010, regarding the protection of animals used for scientific purposes, the Italian legislation (D. Lgs. n. 26/2014) does not require approval from ethics committees for the use of samples submitted or taken for diagnostic purposes. When submitting samples, clinicians and owners signed an informed consent statement for samples to be used for research. No additional biopsies were performed at surgery specifically for this study.

To facilitate the comparison with other existing ACRs and with human cancer registries, all cases received and analyzed were classified following the World Health Organization (WHO) International histological classification of tumors of domestic animals as previously described [25] and coded according to topographical and morphological codes of the WHO’s International Classification of Disease for Oncology system (ICD-O-3.2) [26].

All information collected from the form, as well as data regarding tumor diagnoses and topography, was uploaded and digitalized through a laboratory information system. This system is updated with new cases every six month and can extract data in an Excel file.

### 2.2. Data Handling and Preparation

All data collected by the laboratory information system were extracted and further analyzed in an Excel file format. Each record of the file corresponds to one sample and contains all the information contained in the request form and data on the tumor as assigned by the pathologists (e.g., morphological diagnosis, diagnosis code, topographical code). Information on species, breed, age, topographic location of the tumor and clinical and histological characteristics of tumors were systematically recorded, collected and subsequently used for epidemiological analysis

The age was determined by calculating the difference between the date of birth and the date the tumor was surgically removed. In dogs, the age at diagnosis was categorized into seven age classes (0–3 years, 4–5 years, 6–7 years, 8–9 years, 10–11 years, 12–13 years and >14 years), while eight age classes were considered in cats, with the further addition of the 14–15 years and >16 years classes.

All cases were classified according to the municipality, the province and the region of residence of the animal’s owners. These data were further transferred and analyzed through a geographic information system (GIS) that allows the creation of maps and the spatial distribution of tumor cases in dogs and cats.

Breeds were further classified into two groups both for dogs (purebred, not purebred) and for cats (European, other breeds). The tumor location was obtained by grouping the topographical codes into 14 groups according to Gruntzig et al. [27], except for tumors of the skin (C 44) that were grouped along with tumors of peripheral nerves and autonomic nervous tissues (C 47) and soft tissue neoplasia (C 49) in one category (Table 1).

Tumor diagnoses and malignancy grade were grouped according to Gruntzig et al. [27] following the ICD-O-3.2 classification codes (Table 2); each tumor group was divided into benign (behavior code /0: for benign tumors) and malignant (behavior /1: for uncertain or borderline malignancy; /2: for in situ neoplasms and malignant; /3: malignant tumors) as previously described [28]; tumors coded with /1 were categorized as shown in Table 3 [28]. Cutaneous plasma cell tumors without malignant features were coded as benign with /0 code.

With repeated records of the same tumor in the same animal and in the same location (e.g., two or more squamous cell carcinomas detected in the skin), only one tumor record was counted. If the same subject was diagnosed with more than one tumor type, these were registered as separate records. Metastasis and relapsed tumors (e.g., same tumor type that recurs in the same animal and in the same location after complete surgical excision) were excluded from the analysis, as well as hyperplastic and preneoplastic lesions. Tumors with unspecified or uncertain benign or malignant diagnosis were grouped under the “unspecified tumors” group (ICD-O 8000).

### 2.3. Statistical Analyses

A descriptive analysis of the age at the time of diagnosis, sex, reproductive status, place of residence, tumor location and tumor diagnoses was performed.

Due to the unavailability of the canine and feline population data for Abruzzo and Molise regions, i.e., the denominator, to yield the overall proportion of tumors malignant, as well as the proportion among dogs and cats, the number of cases for a specific category of the tumors in the registry was divided by all submitted tumors. The proportional morbidity (PM) and relative 95% confidence intervals (95% CI) were therefore used as a measure of occurrence, as previously described [25,29]. The proportional morbidity ratio (PMR) was instead chosen as an approximation of the relative risk to make comparisons between groups. More in detail, the PMR was determined by calculating the ratio between the PM of two distinct populations with different exposure levels (e.g., the PM of malignant mammary tumors in spayed females compared to the PM of malignant mammary tumors in intact females, where the category is the tumor location and the populations being compared are spayed versus intact females). PMR was calculated by means of Poisson regression models for different variables such as sex and neutered/spayed status, breed (purebred/mixed), feeding habits (homemade/commercial) and living environment type (indoor/outdoor) [29]. To calculate the PMRs, statistical analyses were performed using R (version 4.0.2, R Foundation for Statistical Computing, Vienna, Austria), while GraphPad Prism 10.2 version was used to graphically visualize the results.

## 3. Results

### 3.1. Dataset

**Figure 1:**
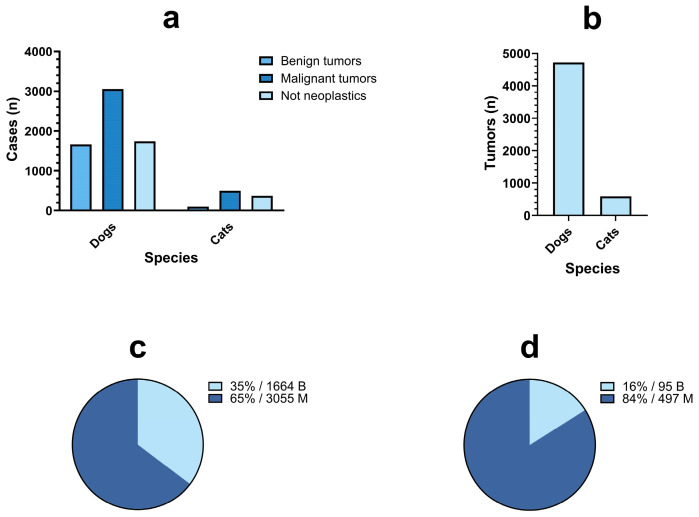
(**a**) Number and percentage of cases (organs and biopsy samples) received by the laboratory-based cancer registry of Abruzzo and Molise regions between 2014 and 2023 divided by type of diagnosis (benign tumors, malignant tumors, not neoplastic/other diseases) and by species. (**b**) Number and percentage of cases diagnosed as tumor by species from 2014 to 2023. (**c**,**d**) Percentage of benign tumors (letter B indicates benign tumors, azure color) and of malignant tumors (letter M indicates malignant tumors, blue color) in dogs (**c**) and cats (**d**).

During the first 10 years of activity, the pathology-based animal cancer registry of Abruzzo and Molise regions received and analyzed 7434 histological samples (organs and biopsy) sent by 111 different veterinary clinics and/or practitioners located in Abruzzo and Molise regions. Of those samples, 5311 (71.5%) were diagnosed as tumors (n = 4719/5311, 88.8% in dogs and n = 592/5311, 11.2% in cats) while 2113 samples (28.5%) were not neoplastic (n = 1740/2113, 82.3% in dogs and n = 373/2113, 17.7% in cats). In dogs, 1664/4719 (35.3%) samples were diagnosed as benign tumors and 3055 (64.7%) were diagnosed as malignant. In cats, the proportion was quite different, with 95/592 tumors that were benign (16%) and 497 tumors classified as malignant (83.9%) (Figure 1a–d). Tumors that were diagnosed post-mortem during diagnostic or forensic autopsy since 2021 were 53/5311 (1%). Number of tumors, mean age at first diagnosis by species, sex, reproductive status and tumor behavior are summarized in Table 4. For both dogs and cats, the majority of tumors were diagnosed in females (n = 3177/4719, n = 360/592, 67.3% and 61.2%, respectively). Most of the female dogs and cats were spayed (n = 1814/4719, 266/592, 38.4% and 45.1%, respectively). The majority of the male dogs were non-neutered (n = 1299/4719, 27.5%), while most of male cats were neutered (143/592, 24.3%). The mean age at first diagnosis showed not significant differences when considering neutering status and cancer behavior. Both for benign and malignant tumors, neutered/spayed dogs and cats were slightly older at first diagnosis than entire animals, and malignant tumors were firstly diagnosed in slightly older subjects. When a tumor was diagnosed (benign or malignant), cats were slightly older than dogs.

### 3.2. Geographic Distribution

**Figure 2:**
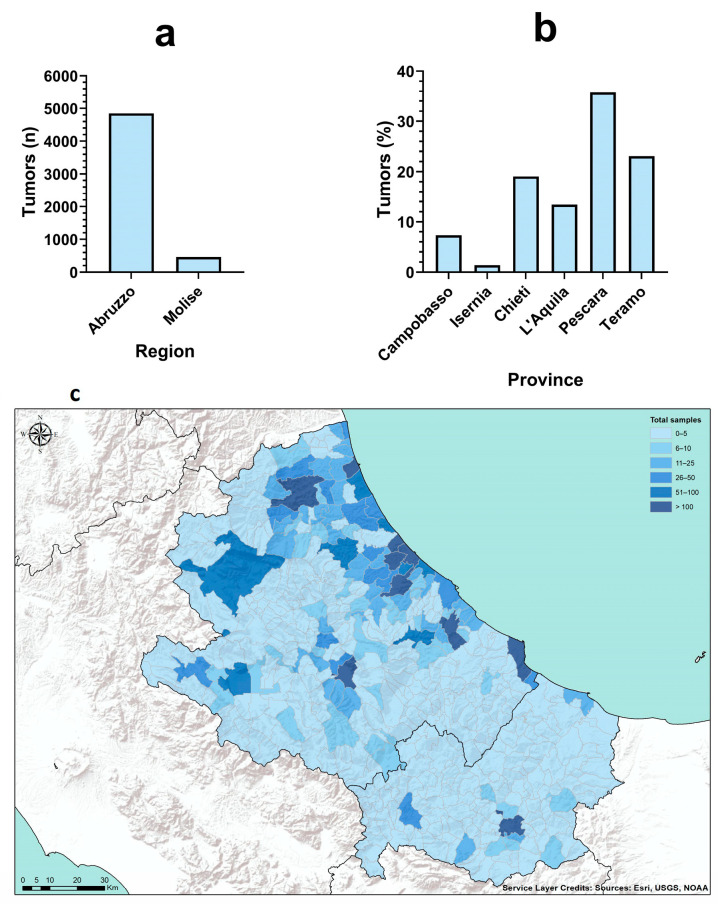
Number of tumor diagnoses in dog and cat by region (**a**) and province (**b**) of residence of the animals/owners. (**c**) Choropleth maps showing the number of dogs and cats included in the study for each municipality of residence of the animals/owners. The gradient of color is proportional to the frequency of observations (from 0 to >100 neoplastic cases). The darker the color, the higher the frequency. Tumor frequency was divided into 6 quantiles (0–5, 6–10, 11–25, 26–50, 51–100 and >100 tumor cases).

Most tumors were collected from dogs and cats residing in the Abruzzo region (n = 4848/5311, 91.3%), while only 463/5311 tumors (8.7%) were recorded from the Molise region (Figure 2a). The most represented province was Pescara (n = 1898/5311, 35.7%), followed by Teramo and Chieti (n = 1277/5311, 23.1% and n = 1011/5311, 19%, respectively). The province of Isernia was the least in terms of tumors frequency, with only 1.4% (n = 74/5311) of diagnoses (Figure 2b). Figure 2c is a choropleth that shows the number of dogs and cats included in the study for each municipality of residence of the animal’s owner. The vast majority of tumor cases belonged to highly populated municipalities near the seacoast or to the most populated province’s administrative centers. Tumor cases were derived from 284 municipalities out of a total of 441. Municipalities that registered 0 to 5 tumor cases were 173, while >100 tumor cases were collected from 11 municipalities. In particular, the municipalities of Pescara, Montesilvano and Teramo recorded 667/5311 (12.6%), 400/5311 (7.5%) and 361/5311 (6.8%) tumor cases, respectively.

### 3.3. Age Distribution

**Figure 3:**
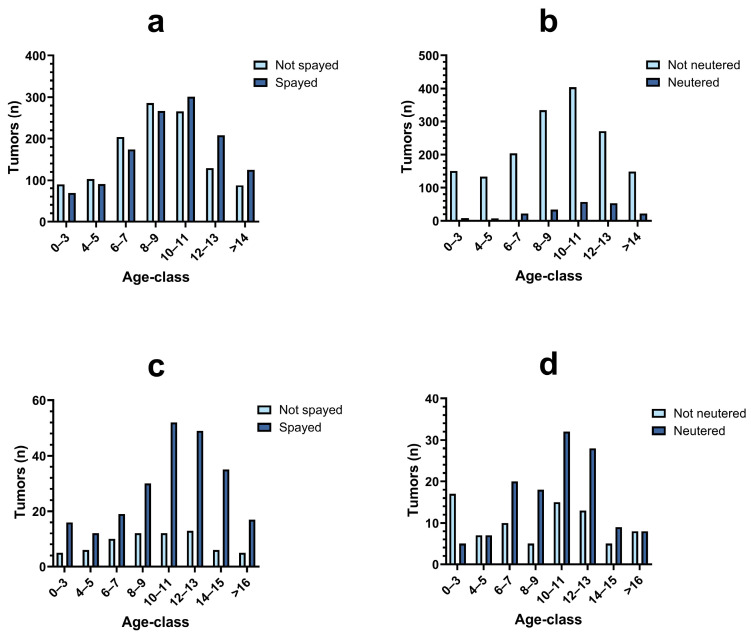
Number and distribution of tumor cases by sex, reproductive status and age class. (**a**) Female dogs; (**b**) male dogs; (**c**) female cats; (**d**) male cats.

The age distribution of the cases (Figure 3) showed a progressive increase in tumor frequency with age. The most represented age classes were between 6 and 13 years for dogs (n = 3419/4719, 72.5%) and between 6 and 15 years for cats (n = 393/592, 66.4%) regardless of the sex. In male dogs, the non-neutered status was the most represented in all age classes, while spayed females were the majority in the age group 10 to >14 years. In cats, neutered and spayed subjects were the most represented in all age classes except for the group 0–3 years male cats, where the majority was non-neutered.

### 3.4. Breed Distribution and Malignancies in Purebred Dogs

**Figure 4:**
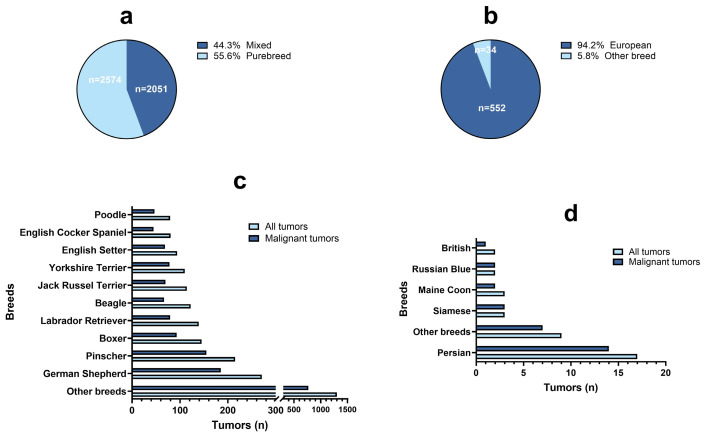
Percentage and number of mixed and purebred dogs (**a**) and European and purebred cats (**b**) with a diagnosis of tumor. (**c**) The 10 most common dog breeds out of 113 breeds with a diagnosis of tumor (azure color) and malignant tumor (blue color). (**d**) The 5 most common cat breeds out of 12 breeds (European breed excluded) with a diagnosis of tumor (azure color) and malignant tumor (blue color).

The dataset included 113 different canine and feline breeds (n = 5211). In dogs, the majority of neoplastic cases were reported in purebreds (n = 2574/4625, 55.7%), with 44.3% (n = 2051/4625) in mixed breeds or cross breeds. The feline breed with the highest frequency of tumors was the European (n = 552/586, 94.2%), with only 5.8% of cases observed in other purebreds (n = 34/586). The most commonly affected dog breed was German shepherd (n = 271/2574, 10.5%), followed by Pinscher (n = 215/2574, 8.4%), Boxer (n = 145/2574, 5.6%) and Labrador retriever (n = 139/2574, 5.4%). The feline purebred with the highest frequency of cases was Persian (n = 17/34, 50%). In both species, there were no differences in terms of proportion of malignant tumors between breeds (Figure 4). For 100 tumors, the breed was not reported (n = 94 for dogs, n = 6 for cats).

**Figure 5:**
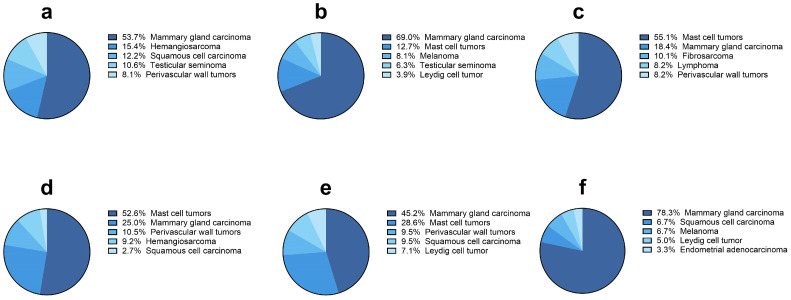
The five most frequent diagnosis of malignant tumors in the six most common dog breeds with a diagnosis of malignancy. (**a**) German shepherd; (**b**) Pinscher; (**c**) Labrador retriever; (**d**) Boxer; (**e**) Beagle; (**f**) Yorkshire terrier.

Figure 5 shows the five most frequent malignant tumors in the six most common dog breeds. Mammary gland carcinomas were the most frequent malignant tumors except for in Labrador retriever and Boxer, where mast cell tumors were more frequently detected (n = 27/49, 55.1% and n = 40/76, 52.6%, respectively). Yorkshire terrier and Pinchers showed a large proportion of mammary carcinomas compared to other breeds, with 47/60 (78.3%) and 87/126 (69%) cases, respectively, followed by German shepherd (n = 66/123, 53.7%) and Beagle (n = 19/42, 45.2%).

### 3.5. Location of Tumors by Species, Sex and Reproductive Status

**Figure 6:**
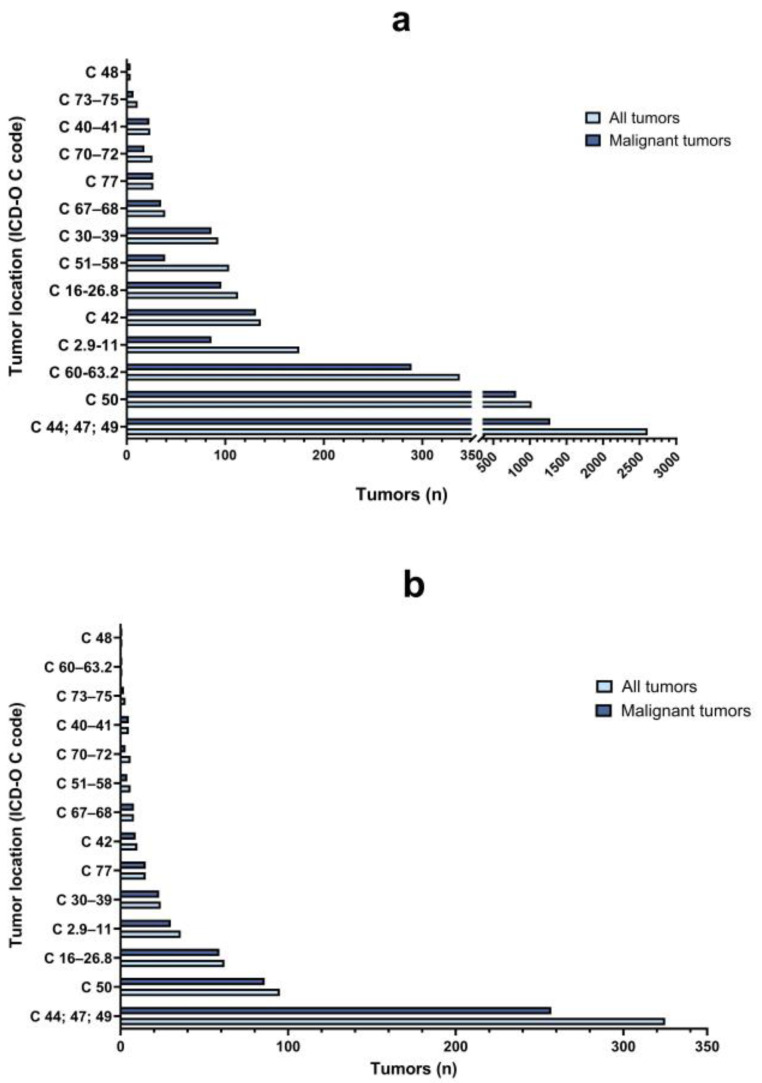
Frequency (n) of total tumors (azure color) and malignant tumors (blue color) in dogs (**a**) and cats (**b**) by topography (tumor location is coded with ICD-O C code; see Table 1 for further details).

As shown in Figure 6, canine tumors were predominantly located in the skin and subcutaneous tissues (n = 2609/4719, 55.3%), followed by mammary gland (n = 1024/4719, 21.7%) and male sexual organs (n = 338/4719, 7.2%). In cats, most tumors were detected in the skin and subcutaneous tissues (n = 325/592, 54.9%), mammary gland (n = 95/592, 16%) and gastrointestinal tract (n = 62/592, 10.5%). Regarding the frequency of malignant tumors in specific locations, in dogs, a higher number of cases was observed in the blood and hematopoietic system (n = 131/136, 96.3%), lymph nodes (n = 27/27, 100%), bones, joints and cartilage (n = 23/24, 95.8%) and respiratory systems and intrathoracic organs (n = 86/93, 92.5%). In cats, the malignancy rate was higher than in dogs with the exception of tumors located in the blood and hematopoietic system (n = 9/10, 90%) and tumor of brain, meninges, eyes and other parts of the CNS (n = 3/6, 50%).

### 3.6. Frequency of Tumor Diagnoses

**Figure 7:**
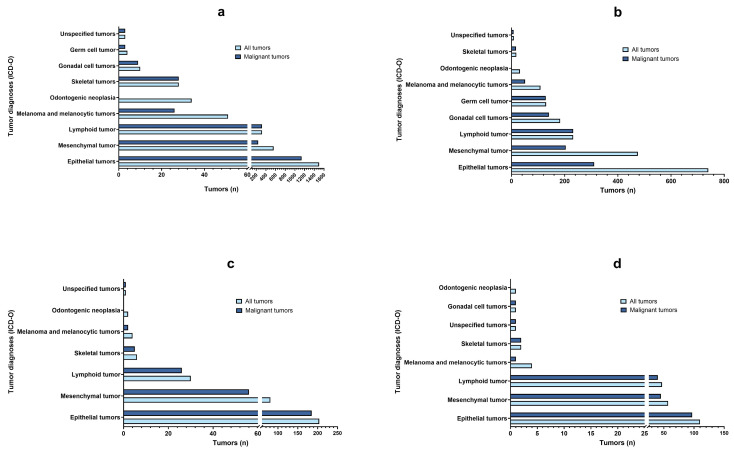
Frequency (n) of tumor diagnoses (all tumors = azure color; malignant tumors = blue color) in dogs (**a**,**b**) and cats (**c**,**d**). (**a**,**c**) Females; (**b**,**d**) males.

The most common tumor diagnoses in dogs and cats were epithelial (n = 1500/3177, 47.2% in female dogs, n = 740/1542, 48% in male dogs, n = 204/360, 56.7% in female cats, n = 110/232, 47.4% in male cats) followed by mesenchymal (n = 491/3177, 15.4% in female dogs, n = 354/1542, 23% in male dogs, n = 81/360, 22.5% in female cats, and n = 57/232, 24.6% in male cats) and lymphoid (n = 387/3177, 12.2% in female dogs, n = 352/1542, 22.8% in male dogs, n = 30/360, 8.3% in female cats, n = 47/232, 20.3% in male cats), regardless of sex. The fourth most frequent tumors were gonadal cell tumors in male dogs (183/1542, 11.8%), melanoma and melanocytoma in male cats and female dogs (n = 4/232, 1.7% and n = 51/3177, 1.6%, respectively) and skeletal tumors in female cats (n = 6/360, 1.7%). In female dogs and cats, epithelial (n = 1139/1500, 75.9% and n = 185/204, 90%) and mesenchymal neoplasms (n = 241/559, 43.1% and n = 56/81, 69.1%) were more frequently malignant than in male dogs and cats (n = 311/740, 42% and n = 97/111, 87.4% considering epithelial tumors; n = 204/475, 42.9 and n = 37/57, 64.9% considering mesenchymal tumors) (Figure 7).

**Figure 8:**
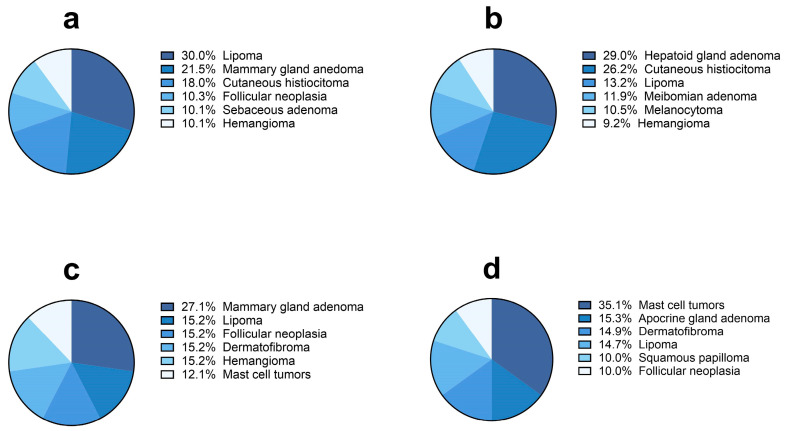
Most frequent six benignant tumor diagnoses (%) in dogs ((**a**), female; (**b**), male) and cats ((**c**), female; (**d**), male).

**Figure 9:**
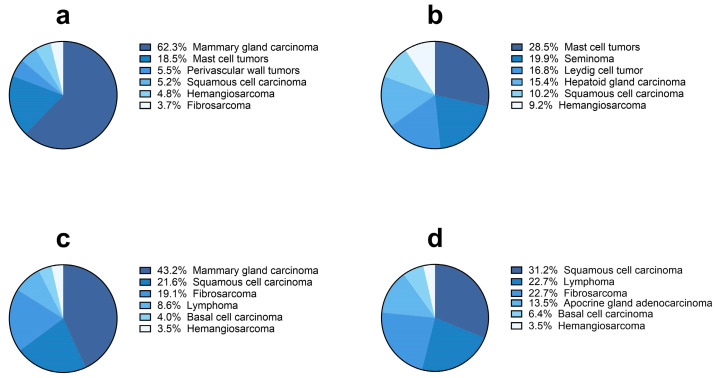
Most frequent six malignant tumor diagnoses (%) in dogs ((**a**), female; (**b**), male) and cats ((**c**), female; (**d**), male).

When considering the six most frequent specific tumors (Figure 8 and Figure 9), lipoma (n = 113/377, 30%), hepatoid gland adenoma (n = 132/455, 29%) and mammary gland adenoma (n = 9/33, 27.1%) were the most represented benign tumors in male and female dogs, respectively, while mast cell tumors (n = 7/20, 35%) and mammary gland adenoma (n = 9/33, 27%) were more frequently detected in male and female cats. Within the category of malignant tumors, mammary gland carcinoma was the most frequent neoplasia diagnosed in female dogs (n = 880/1412, 62.3%) and cats (n = 86/199, 43.2%), while mast cell tumors (n = 185/649, 28.5%) and squamous cell carcinomas (n = 44/141, 31.2%) were more frequently detected in male dogs and cats, respectively.

**Figure 10:**
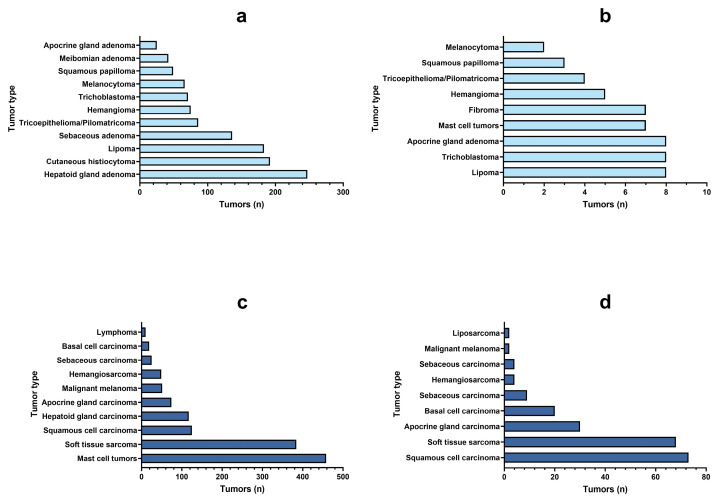
Most frequent skin and subcutaneous (C 44; 47; 49) benign tumor diagnoses in dogs and cats ((**a**) and (**b**), respectively). Most frequent malignant skin and subcutaneous (C 44; 47; 49) tumor diagnoses in dogs and cats ((**c**) and (**d**), respectively).

Since skin and subcutaneous tumors represented the majority of diagnoses in both species, in Figure 10 are detailed the most frequent benign and malignant tumor morphologic diagnoses affecting this topography. Hepatoid gland adenoma, cutaneous histiocytoma and lipoma were the three most common benign diagnoses in dogs, while lipoma, trichoblastoma and apocrine adenoma were more frequently detected in cats. Mast cell tumors, soft tissue sarcomas and squamous cell carcinomas were the most common canine malignant skin tumors, while squamous cell carcinomas, soft tissue sarcomas and apocrine gland carcinomas predominated in the feline species.

### 3.7. Analysis of Proportional Morbidity Ratios

**Figure 11:**
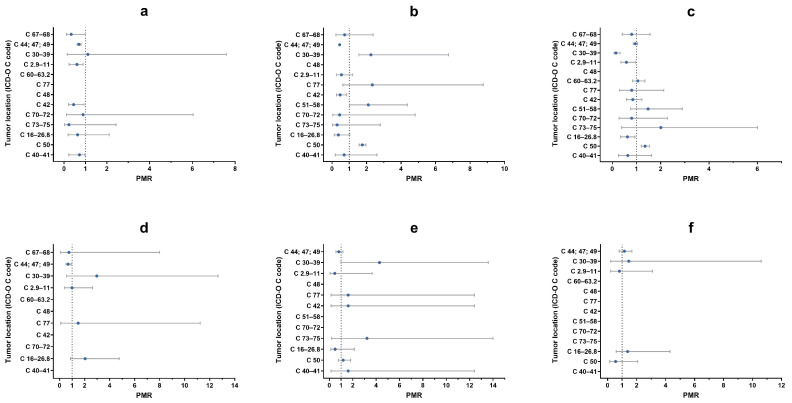
Proportional morbidity ratios (PMRs) of malignant tumors divided by topography, comparing neutered males with non-neutered males ((**a**), dogs; (**d**), cats), spayed females with non- spayed females ((**b**), dogs; (**e**), cats), purebreds dog with mixed dogs (**c**) and European cats with other breeds of cats (**f**). Azure dots represent PMRs. Gray bars represent lower and upper limit of 95% confidence interval. A vertical reference dotted line indicates PMR = 1.

The assessment of proportional morbidity ratios (PMRs) for malignant tumors in relation to topography highlighted some “risk and/or protective” factors that could influence the development of certain type of tumors (Figure 11). The risk of developing tumors of the blood and hematopoietic system (PMR = 0.44; 95% CI: 0.21–0.94), skin and subcutaneous tissues (PMR = 0.70; 95% CI: 0.61–0.80), oral cavity and pharynx (PMR = 0.60; 95% CI: 0.24–0.89), urinary organs (PMR = 0.33; 95% CI: 0.11–0.99) and bones, joints and cartilage (PMR = 0.72; 95% CI: 0.22–0.98) was lower in non-neutered male dogs than in neutered male dogs (Figure 11a). Non-spayed female dogs had a greater risk of developing tumors of the mammary gland (PMR = 1.75; 95% CI: 1.57–1.96), female sexual organs (PMR = 2.12; 95% CI: 1.01–4.36) and respiratory system (PMR = 2.25; 95% CI: 1.55–6.74) when compared with spayed female dogs. In addition, not being spayed appears to play a protective role against the development of cutaneous and subcutaneous tissues tumors (PMR = 0.44; 95% CI: 0.38–0.51) and blood hematopoietic system tumors (PMR = 0.47; 95% CI: 0.26–0.85) (Figure 11b).

Purebred dogs had a significantly greater risk of developing mammary gland tumors (PMR = 1.36; 95% CI: 1.20–1.54) compared with mixed breed, while being purebred seems to be a “protective factor” for the onset of respiratory system (PMR = 0.15; 95% CI: 0.07–0.32), gastrointestinal tract (PMR = 0.63; 95% CI: 0.34–0.94) and oral cavity (PMR = 0.59; 95% CI: 0.36–0.96) neoplasia (Figure 11c).

Non-neutered male cats had a lower risk of developing skin and subcutaneous tumors (PMR = 0.68; 95% CI: 0.50–0.92) compared with neutered cats, as observed in dogs (Figure 11d).

No significant differences were observed by comparing spayed with non-spayed female cats and other purebred with European cats (Figure 11d,e).

**Figure 12:**
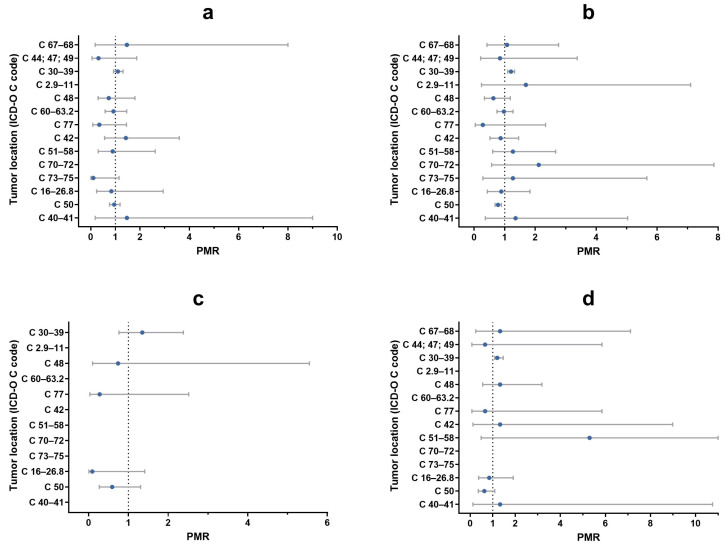
Proportional morbidity ratios (PMRs) of malignant tumors divided by topography, comparing animals with a prevalent homemade diet vs. commercial one ((**a**), dogs; (**c**), cats) and animals that live mainly indoors vs. outdoors ((**b**), dogs; (**d**), cats). Azure dots represent PMRs. Gray bars represent lower and upper limit of 95% confidence interval. A vertical reference dotted line indicates PMR = 1.

The type of diet (commercial vs. home-based diet) did not affect the risk of developing tumors since no significant differences were observed in either species (Figure 12a,c).

The risk of developing skin and subcutaneous tissues tumors was higher for dogs and cats that lived mostly outdoors (PMR dogs = 1.21; 95% CI: 1.10–1.33; PMR cats = 1.18; 95% CI: 1.08–1.47), while dogs that live mainly indoors had a greater risk to develop mammary gland tumors (PMR = 0.78; 95% CI: 0.68–0.89) (Figure 12b,d).

## 4. Discussion

Cancer remains one the most significant causes of mortality and morbidity in humans and is widely considered the foremost global public health challenge of the 21st century [1]. Recently, there has been a noticeable rise in cancer cases among companion animals, which has led to increased research activity and interest within the scientific community [1,15]. While improved veterinary care, advanced diagnostic tools and greater life expectancy may contribute to these rising numbers, the impact of environmental conditions and risks on animal health should not be overlooked. It is now well recognized that animals can serve as sentinels for environmental risk factors due to their shorter lifespan and lower required exposure doses. Additionally, veterinary epidemiological studies offer several advantages, including lower costs and easier access to tissue samples and autopsy data [8,11].

This retrospective study presents data on 5311 tumors collected over ten years from the pathology-based animal cancer registry of the Abruzzo and Molise regions (central Italy). We strongly believe that examining the frequency of naturally occurring tumors in canine and feline populations is crucial in cancer research, as these animals provide a relevant, accessible and reliable model for understanding cancer biology [13,15]. Moreover, findings described herein highlight the fundamental role of cancer registration in advancing veterinary oncology, supporting comparative oncology and contributing to the One Health approach in cancer research.

Our study found that tumor frequency increases with age, peaking in middle/older age groups, which is expected given the cumulative exposure to risk factors over time and the biological aging process [1,30]. The age at first diagnosis for dogs and cats was similar to that reported in previous studies [14,17,27,28], with cats developing tumors about 6 months later than dogs, possibly due to their longer lifespan.

Particularly in cases of malignancy, neutered and spayed dogs and cats presented with tumors at a slightly older age compared to their non-neutered or non-spayed counterparts. Additionally, tumors were more frequently detected in female dogs and cats than in male, a finding consistent with several previous studies [17,28,29,30,31,32,33,34], suggesting a potential influence of sex on tumor development and progression [28]. However, these observations may also be due to the higher occurrence of sex-specific tumors and should not be generalized. Notably, mammary gland tumors were the second most frequent in both dogs and cats, with a higher incidence in non-spayed females, corroborating the established link between reproductive status and certain type of neoplasia.

Overall, a higher frequency of malignant tumors was detected in cats compared to dogs, suggesting a more aggressive cancer profile in this species. Malignant tumors were more prevalent in specific anatomical sites, such as the blood and hematopoietic system, lymph nodes and bones in dogs and the mammary gland and skin in cats. Similar results were observed by Pinello et al. in Portugal [30]. This cross-species variation could be explained by the higher cancer mortality risk observed in *Carnivora*, linked with the consumption of mammalian prey, a feature especially pronounced in obligatory carnivores like cats [35].

Most tumor cases were diagnosed in dogs and cats living in densely populated areas near the seacoast and major urban centers, reflecting the distribution of veterinary clinics and/or the high number of pets in such areas. Conversely, the lower frequency of tumors in rural areas may be attributed to underreporting, limited access to diagnostic facilities and smaller canine and feline populations. These differences could also be partially attributed to the presence of high environmental pollution typically found in heavily urbanized areas [1,8]. Further studies are recommended to investigate the association between environmental pollution and the presence of carcinogenic substances in water, soil and food in districts with a high incidence of malignant cases and/or specific tumors.

It is widely acknowledged that significant differences exist between dog breeds in their risk of developing particular types of tumors [36]. In our survey, the frequency of tumors and the proportion of malignant ones appeared higher in purebred dogs compared to mixed breed. However, no differences were detected in terms of the proportion of malignant tumors between breeds. More data are needed to further investigate the incidence rates of different tumors across purebreds. Among the six most frequent breeds in our database, excluding mammary carcinomas, Boxers and Labrador retrievers showed a higher frequency of mast cell tumors, German shepherds demonstrated a higher frequency of hemangiosarcomas and Pinschers a higher frequency of melanomas. These findings align with the existing literature [36,37,38,39] and emphasize the genetic predisposition of certain dog breeds to specific types of cancer. Nevertheless, it is important to recognize that the etiology of most tumors is multifactorial, and while genetics play a significant role, epigenetics, environmental influences and hormonal/metabolic factors also contribute to the risk of developing certain tumors [36].

In cats, there are fewer genetically distinct breeds compared to dogs [40], and our database contains only 34 tumors from 5 purebred categories, making it difficult to draw conclusions about breed predisposition.

As reported in several studies [32,34,36,41,42], mammary gland tumors are the most frequent in females, while skin tumors are more common in males. Regardless of sex, tumors were predominantly diagnosed in the skin and soft tissues, followed by mammary gland and sexual organs [27,28,33]. This suggests that tumors located in the mammary gland, skin, subcutaneous tissues and genital areas are typically more easily detected by both owners and clinicians during physical exams compared to those in internal organs, which usually require more specialized and expensive diagnostic procedures.

As also reported by Grüntzig et al. [27], the most common tumor diagnoses were epithelial, followed by mesenchymal and lymphoid, regardless of species and sex. The fourth most frequent were gonadal cell tumors in male dogs, melanoma and melanocytic tumors in male cats and female dogs and skeletal tumors in female cats. In female dogs, epithelial neoplasia was more frequently malignant than in male dogs, while in cats, the proportion was quite similar between sexes. This could be related to the high number of mammary gland tumors in females, which are often malignant. In addition, in male dogs, hepatoid gland adenoma, a benign hormonal-related tumor, was frequently detected, thus contributing to the lower proportion of malignant tumors [43].

Our data indicate that skin tumors are the most common in both species, with dogs showing a lower tendency toward malignancy. Primary risk factors for skin tumors in both species include UV radiation, viral infections and immunological status [44]. Notably, UV exposure has been strongly linked to the development of squamous cell carcinomas, especially in light-colored cats. Additionally, there is growing evidence suggesting that papillomaviruses may contribute to the onset of cutaneous squamous cell carcinomas and basal cell carcinomas in cats [44,45,46].

Our study confirms variations in histotype and tumor by malignancy, species and sex, as well as differences based on reproductive status, breed, predominant diet and living environment. Due to the lack of exact data on the reference population of dogs and cats in the Abruzzo and Molise regions (denominators) and the incomplete tumor collection (numerators), we could not determine cancer incidence rates and had to work with proportions instead. Consequently, to compare our dataset and compute association measures, we used proportional morbidity ratios (PMRs). While PMRs are useful for exploratory analysis, they should be interpreted with caution, as their denominator is the total number of cases, not the population at risk, which limits external validity and comparison [47]. Additionally, PMRs allow for direct comparison with results in the literature only if those results are also presented as proportions. Our study highlights certain risk and “protective” factors for the development of tumors in specific topographies. For example, in non-spayed female dogs, there is an increased risk for sex-specific and hormonal-related tumors (e.g., mammary gland and reproductive organs). On the other hand, being intact appears to have a protective influence against malignant tumors affecting the skin, soft tissues, oral cavity, pharynx, urinary organs, bones, joints and cartilage. Further research is necessary and strongly recommended to better examine the impact of reproductive status on various tumor types, including hemangiosarcoma, osteosarcoma, lymphoma, transitional cell carcinoma and mast cell tumors [48,49].

As also reported by other similar studies [28,32,36], our survey found that purebred dogs had a higher risk of malignant mammary tumors than mixed breed dogs, with Pinscher, German shepherd and Yorkshire terrier being more inclined to develop these type of tumors. Although this may not directly mirror the situation in humans, purebreds could serve as a valuable model for studying the influence of genetic traits compared to environmental risk factors in cancer onset and development [32,36].

Chronic exposure to UV radiations in animals that prevalently live outdoors could explain the higher risk of skin tumors observed in dogs and cats. This risk is likely related to the mutagenic effects of UV radiation, which predispose animals to develop certain types of skin and subcutaneous malignant tumors, such as squamous cell carcinoma and hemangiosarcoma [44,45,50,51]. In addition, dogs living outside the home had a greater risk of developing malignant mammary tumors compared to those living indoors, which could be due to the fact that indoor dogs are more closely monitored by their owners, who may pay greater attention to their overall health and well-being.

One of the main pitfalls of this study is the misdiagnoses of tumors that may potentially be present. In addition, there could be present certain limitations that are typical of long-term retrospective studies. One such issue is that diagnoses were made by different pathologists at various time periods. The criteria for some tumor diagnoses may have changed over time, and in some cases, there may be a subjective element in histopathological diagnostics. However, the diagnostic capability of the laboratory and the reproducibility of tumor diagnoses were constantly (every year) verified by the participation with satisfactory results in agreement studies between several Italian laboratories working in the field of diagnostic veterinary oncology (data not published).

The ICD-O codes used for data collection were not tailored for some veterinary tumor entities, as the Vet-ICD-O-Canine-1 [14] was unavailable when data collection began and thus was not utilized. The use of ICD-O classification, which is not fully adapted to veterinary oncology, may lead to imprecision and inaccuracies in the categorization of some animal tumor types. However, the adapted ICD-O classification systems employed in this study were preliminarily agreed upon by pathologists working in our pathology-based ACR, enabling the use of a harmonized classification system and the efficient recording of clear and unambiguous data.

## 5. Conclusions

Data derived from the pathology-based ACR of the Abruzzo and Molise regions, as described and analyzed here, can help identify potential differences in the incidence of certain tumor types. This information allows for the formulation of hypotheses regarding the potential role of specific risk factors in the development of neoplasms, such as geographic location, sex and reproductive status, breed and lifestyle of dogs and cats. These insights may also serve as a catalyst for further studies aimed at identifying and preventing the risk factors most commonly associated with tumor development in pets, with potential implications for human health. Additionally, this work underscores the importance of animal tumor registries in promoting the information exchange and collaboration with human cancer registries, fully supporting the One Health approach.

## Figures and Tables

**Table 1:** Coding of tumor locations according to ICD-O-3.

Location	ICD-O C Code
Blood and hematopoietic system	ICD-O C 42
Bones, joints and cartilage	ICD-O C 40–41
Brain, meninges, eyes and other parts of CNS	ICD-O C 70–72
Mammary gland	ICD-O C 50
Endocrine gland	ICD-O C 73–75
Gastrointestinal tract (excluded oral cavity)	ICD-O C 16–26.8
Lymph nodes	ICD-O C 77
Male sexual organs	ICD-O C 60–63.2
Oral cavity and pharynx	ICD-O C 2.9–11
Female sexual organs	ICD-O C 51–58
Respiratory system and intrathoracic organs	ICD-O C 30–39
Retroperitoneum and peritoneum	ICD-O C 48
Skin and soft tissues (subcutaneous, connective tissue and skeletal muscle)	ICD-O C 44; 47; 49
Urinary organs	ICD-O C 67–68

**Table 2:** Coding and grading of tumor diagnoses according to ICD-O-3.

Diagnoses	ICD-O Code
Odontogenic neoplasia	ICD-O 9270–9330
Epithelial tumors	ICD-O 8010–8587,
	ICD-O 9050–9058
Germ cell tumors	ICD-O 9060–9085
Lymphoid tumors	ICD-O 9590–9960
Melanoma and melanocytic tumor	ICD-O 8720–8790
Mesenchymal tumors	ICD-O 8680–8711,
	ICD-O 8800–9040,
	ICD-O 9120–9175,
	ICD-O 9580
Skeletal tumors	ICD-O 9180–9262
Neural tumors	ICD-O 9380–9570
Gonadal tumors	ICD-O 8610–8670
Unspecified tumors	ICD-O 8000

**Table 3:** Assessment of tumors with /1 behavior code.

Diagnosis	Malignancy category
Canine cutaneous MCT (all grades)	Malignant
Feline cutaneous MCT	
- Without malignancy description	Benign
- With malignancy description	Malignant
Subcutaneous MCT	Benign
Meibomian gland epithelioma	Malignant
Acanthomatous ameloblastoma	Benign
All /1 morphologies	
- Without malignancy description	Benign
- With malignancy description	Malignant

**Table 4:** Number and percentage of tumors and mean age at first diagnosis by species, sex, neutering status and tumor behavior.

	Sex and Neutering Status	Number (n)	Percentage (%)	Mean Age at Diagnosis (Years) (Benign Tumors)	Mean Age at Diagnosis (Years) (Malignant Tumors)
**Dogs**	Male neutered	243	5.2%	9.4	10.3
	Male non-neutered	1299	27.5%	8.9	9.7
	Female spayed	1814	38.4%	9.2	9.8
	Female non-spayed	1363	28.9%	8.4	9.0
	Total	4719	100%	9.0	9.7
**Cats**	Male neutered	143	24.3%	9.7	10.3
	Male non-neutered	89	14.5%	9.1	9.4
	Female spayed	266	45.1%	10.1	11.1
	Female non-spayed	94	16.1%	9.4	9.8
	Total	592	100%	9.6	10.2

## Data Availability

The data presented in this study are available on request from the corresponding author.
